# UCK2 Drives Lung Adenocarcinoma Progression and Immune Dysregulation via the RHEB/mTOR Signaling Axis

**DOI:** 10.32604/or.2026.078651

**Published:** 2026-05-21

**Authors:** Xiaolin Wei, Jing Guo, Chuntao Tao, Yong Bao, Li Yang, Hong Chen

**Affiliations:** 1 Department of Respiratory and Critical Care Medicine, The First Affiliated Hospital, Chongqing Medical University, Chongqing, China; 2 Department of Respiratory Medicine, Sichuan Taikang Hospital, Chengdu, China; 3 Department of Biochemistry and Molecular Biology, College of Basic Medical Sciences, Chongqing Medical University, Chongqing, China

**Keywords:** Uridine-cytidine kinase 2 (UCK2), lung adenocarcinoma (LUAD), prognostic biomarker, RHEB/mTOR signaling, drug sensitivity

## Abstract

**Objectives:** Uridine-cytidine kinase 2 (UCK2) plays a crucial role in the pyrimidine salvage pathway, but its function in lung adenocarcinoma (LUAD) is still largely unclear. The study aimed to investigate the expression, prognostic value, biological functions, and molecular mechanisms of UCK2 in LUAD. **Methods:** Bioinformatic analyses were performed using The Cancer Genome Atlas (TCGA), Gene Set Cancer Analysis (GSCA), Gene Expression Omnibus (GEO), and Genotype Tissue Expression (GTEx) datasets. *In vitro* assays evaluated the effect of UCK2 overexpression on LUAD cells. Co-immunoprecipitation and pathway analyses were utilized to explore the underlying mechanism. Immune landscape and drug sensitivity analyses were also carried out. **Results:** UCK2 was markedly upregulated in LUAD tissues, correlating with advanced tumor stage and poor overall survival, and served as an independent prognostic factor. Functionally, overexpression of UCK2 increased the proliferation and migration of LUAD cells. Mechanistically, UCK2 interacted with the small GTPase Ras homolog enriched in brain (RHEB) and regulated the mechanistic target of rapamycin (mTOR) signaling pathway. UCK2 expression was also associated with specific immune profiles and drug sensitivity in LUAD. **Conclusion:** UCK2 acts as a prognostic indicator and oncogene in LUAD, at least partly via the RHEB/mTOR axis. Targeting UCK2 may represent a promising therapeutic approach for LUAD.

## 1 Introduction

Lung cancer (LC) is the foremost cause of cancer-related deaths globally, with lung adenocarcinoma (LUAD) being the most prevalent histological subtype, accounting for approximately 40% of all LC cases [1,2]. According to recent global cancer statistics, the incidence and mortality of LUAD continue to rise, representing a substantial public health burden worldwide [3,4]. Although therapeutic modalities have advanced—encompassing surgery, platinum-based chemotherapy, targeted medicines against driver mutations like epidermal growth factor receptor (EGFR) and immune checkpoint inhibitors (ICIs)—the clinical outcomes for LUAD patients remain suboptimal [5]. A significant clinical challenge is the frequent diagnosis of LUAD at advanced, metastatic stages. Notably, distant metastasis can occur even when the primary tumor is small [6], severely constraining effective treatment options. Moreover, the emergence of acquired resistance to targeted therapies and the heterogeneous response to ICIs further diminish clinical benefits [7]. Comprehensive multi-omics integration has emerged as a key approach to uncover novel biomarkers and therapeutic opportunities in oncology [8]. Against this backdrop, the absence of robust biomarkers to reliably predict outcomes and guide therapy selection underscores the critical need to delineate the key molecular drivers underlying LUAD progression and metastasis.

Importantly, uridine-cytidine kinase 2 (UCK2) has recently been identified as a possible regulator of malignant behavior in tumors. UCK2 is a cytoplasmic enzyme that serves as a key component of the pyrimidine salvage pathway, facilitating the transformation of uridine and cytidine into uridine monophosphate (UMP) and cytidine monophosphate (CMP), respectively [9]. Beyond this metabolic role, accumulating evidence implicates UCK2 dysregulation in the pathogenesis of various malignancies [10,11,12,13]. For example, it has been demonstrated that UCK2 overexpression promotes cell proliferation and imparts cisplatin resistance in intrahepatic cholangiocarcinoma by activating the phosphatidylinositol 3-kinase (PI3K)/protein kinase B (AKT)/mammalian target of rapamycin (mTOR) pathway, which is a crucial regulator of autophagy [10]. Likewise, increased UCK2 expression corresponds with poor clinical results in melanoma and hepatocellular carcinoma [11,12]. Despite these insights, the landscape of UCK2 in LUAD remains largely uncharted. Its expression profile, correlation with LUAD-specific clinicopathological features (e.g., tumor stage), and functional impact on core malignant phenotypes such as invasion and proliferation are poorly defined. Furthermore, there is a lack of systematic assessment of whether and how UCK2 affects therapeutic responses, including sensitivity to conventional chemotherapy and targeted medicines, which limits the evaluation of its potential as a therapeutic target and clinical prognostic indicator for LUAD.

To bridge these gaps and systematically map the multifaceted role of UCK2 in LUAD, we adopted an integrative multi-omics profiling paradigm. This approach, which has been effectively used to characterize other oncogenic drivers, allows for the concurrent exploration of a gene’s clinical, molecular, and therapeutic landscape. We leveraged this paradigm to: (1) delineate UCK2 expression patterns across tissues and cancer types; (2) evaluate its prognostic and clinicopathological associations; (3) explore its epigenetic regulation; (4) assess its preliminary functional impact via overexpression; and (5) predict its potential influence on therapeutic responses.

Through this systematic profiling, our study aims to establish UCK2 as a pivotal, multi-faceted driver in LUAD and to generate a comprehensive set of hypotheses regarding its mechanisms and translational potential, laying a solid groundwork for future in-depth mechanistic and clinical studies.

## 2 Materials and Methods

### 2.1 Lung Cancer Transcriptomic Datasets

The RNA sequencing (RNA-seq) data and associated clinical characteristics of LUAD samples utilized in this study were obtained from The Cancer Genome Atlas (TCGA) database (https://www.cancer.gov/tcga), which includes 483 primary LUAD tissue specimens and 59 adjacent normal tissue samples [14]. To supplement normal tissue expression signatures, gene expression profiles were obtained from the Genotype-Tissue Expression (GTEx) database (https://commonfund.nih.gov/GTEx), and the Cancer Cell Line Encyclopedia (CCLE) database (https://portals.broadinstitute.org/ccle/) was leveraged to obtain gene expression profiles across a panel of human tumor cell lines. For a thorough assessment of UCK2 mRNA expression patterns, UCK2 expression data from TCGA, Gene Set Cancer Analysis (GSCA) (https://guolab.wchscu.cn/GSCA/#/), and the Gene Expression Omnibus (GEO) (https://www.ncbi.nlm.nih.gov/geo) were combined to analyze its expression across a broad range of tumor types and normal tissues, as well as its correlation with clinicopathological staging. Corresponding DNA methylation data linked to LUAD were also extracted from the TCGA database for integrated multi-omic analysis. For external survival validation, two independent LUAD cohorts from GEO—GSE30219 [15] and GSE68465 [16]—were incorporated alongside the TCGA-LUAD discovery cohort. Multivariate Cox regression analysis and prognostic nomogram construction were applied to the TCGA-LUAD cohort. Patients were included according to predefined inclusion and exclusion criteria. The inclusion criteria were: (1) pathologically confirmed primary LUAD; (2) availability of complete UCK2 mRNA expression data; and (3) complete clinicopathological data (T/N/M stages, age, and gender) linked with overall survival (OS) information. The exclusion criteria were: (1) incomplete data for any of the aforementioned key variables; (2) non-primary LUAD or mixed histological subtypes; and (3) duplicate samples. Following this complete-case analysis, patients with missing data for key variables were excluded, yielding a final analytical cohort of 388 patients. The proportional hazards assumption was validated using Schoenfeld residual analysis (global test *p* > 0.05), which confirmed the validity of the model. Kaplan-Meier survival curves for the TCGA cohort were created using the GEPIA2 online tool (http://gepia2.cancer-pku.cn) with a median cutoff for UCK2 expression, and external survival validation in GEO cohorts was performed using the KM-Plot platform, adhering to standardized analytical protocols [17]. Notably, TCGA and GEO are public, de-identified databases for which informed consent had been secured from all participants in the original studies, with all data accessed in compliance with their respective data usage policies. As this study relied only on publicly available and ethically approved open-source data, no additional ethical approval or institutional review board (IRB) consent was required. No conflicts of interest were declared.

### 2.2 Functional Enrichment Analysis

Gene Ontology (GO) and Kyoto Encyclopedia of Genes and Genomes (KEGG) pathway enrichment analyses were implemented to characterize differentially expressed genes (DEGs) in UCK2 high-expression versus low-expression subgroups, utilizing the R package clusterProfiler (Version 3.6.2, https://bioconductor.org/packages/release/bioc/html/clusterProfiler.html). Concurrently, the ‘fgsea’ R package (Version 1.28.0) was employed to carry out gene set enrichment analysis (GSEA) and screen for pathways with statistically significant enrichment. Thresholds for defining significant DEGs and enriched pathways were set as log2 fold change (log2FC) > 1 and adjusted *p*-value < 0.05.

### 2.3 DNA Methylation Analysis

DNA methylation and gene expression data for the TCGA-LUAD cohort were retrieved from the UCSC Xena database (https://xenabrowser.net). Methylation data were generated using the Illumina HumanMethylation450K BeadChip platform, and preprocessed matrix data (including background correction and quantile normalization) were directly utilized without additional processing. Patients were stratified into UCK2 hypomethylation and hypermethylation subgroups, with the median methylation level of the UCK2 gene serving as the cutoff value. Using the chi-square test or Fisher’s exact test, the relationships between UCK2 expression, DNA methylation, and a variety of categorical clinicopathological variables were evaluated. Spearman’s rank correlation analysis was performed at the level of individual CpG sites to examine the correlation between UCK2 gene expression and its DNA methylation, and Bonferroni correction was applied to address the issue of multiple testing. Additionally, A549 and H1975 cells were incubated with Decitabine (a DNA methyltransferase inhibitor) at a final concentration of 10 μM for 72 h, and quantitative real-time PCR (RT-qPCR) was employed to measure alterations in UCK2 mRNA expression levels. The correlation between distinct UCK2 methylation sites and LUAD prognosis was evaluated using OS as the endpoint. Both Kaplan–Meier survival analysis and multivariate Cox proportional hazards regression (adjusted for age, gender, and TNM stage) were performed to assess the prognostic significance of UCK2 methylation.

### 2.4 Immune Profiling and Gene Expression Analysis

According to their UCK2 expression, patients were divided into high and low expression cohorts. Three established computational tools were employed to characterize the immune landscape: immune cell deconvolution using the Cell-type Identification By Estimating Relative Subsets Of RNA Transcripts (CIBERSORT) method, tumor microenvironment scoring with the Estimation of STromal and Immune cells in MAlignant Tumor tissues using Expression data (ESTIMATE) algorithm, and immune cell activity evaluation through single-sample Gene Set Enrichment Analysis (ssGSEA). For ssGSEA, immune gene sets were obtained from the Molecular Signatures Database (MSigDB) C7 collection, which comprises immunologic signature gene sets that represent various immune cell types, pathways, and functions. Variations in immune infiltration among algorithms were illustrated using heatmaps. Statistical comparisons of immune cell infiltration estimate between high- and low-expression groups of UCK2 were conducted using the Wilcoxon rank-sum test. No additional analysis was performed to assess the concordance among the results generated by different algorithms. A concomitant investigation was conducted to compare the expression profiles of immune checkpoint genes between the two patient cohorts. To account for multiple comparisons in immune-related analyses, Bonferroni correction was applied to adjust *p*-values and control the family-wise error rate. To evaluate UCK2’s potential role in immunotherapeutic strategies, predictive modeling of immune checkpoint inhibitor (ICI) responsiveness was performed using the Tumor Immune Dysfunction and Exclusion (TIDE) tool. This approach integrates TIDE-associated features into a tumor immune evasion framework to generate ICI response predictions.

### 2.5 Evaluation of Drug Sensitivity

To investigate the link between UCK2 levels and LUAD responsiveness to therapeutic interventions, we applied the pRRophetic method (Version 0.5) to forecast the half-maximal inhibitory concentration (IC_50_) of chemotherapeutic agents as well as targeted therapeutics. This predictive analysis was based on the development of a ridge regression model that incorporated gene expression profiles from the Genomics of Drug Sensitivity in Cancer (GDSC) database (https://www.cancerrxgene.org/) and the TCGA-LUAD cohort. We initially computed the correlation coefficient between the drug IC_50_ value and UCK2 gene expression in order to analyze the relationship between UCK2 expression and anti-tumor drug sensitivity. Drugs with a correlation coefficient > 0.2 and a *p*-value < 0.05 were selected for subsequent in-depth analysis, including AG014699, AZD6244, AZD6482, BID1870, CGP082996, Docetaxel, Elesclomol, Epothilone B, GSK269962A, GSK650394, JNK Inhibitor VIII, KU55933, LFMA13, MG132, NU7441, Obatoclax Mesylate, Parthenolide, Pazopanib, PF562271, PF4708671, QS11, RDEA119, SL01011, Thapsigargin and TW37. Notably, a higher IC_50_ value denotes diminished sensitivity of LUAD cells to the corresponding agent, which reflects a more prominent drug-resistant phenotype.

### 2.6 Cell Lines and Culture Conditions

We acquired human LUAD cell lines A549 (SCSP-503), H1975 (SCSP-597), and PC9 (SCSP-5085) from the Cell Bank of the Chinese Academy of Sciences (Shanghai, China). The gefitinib-resistant PC9 subline (PC9 GR, YS4389C) and osimertinib-resistant H1975 subline (H1975 OR, CTCC-0281-NY) were acquired from Shanghai Yaji Biological Technology Co., Ltd. (Y-J Biological, Shanghai, China) and Zhejiang Meisen Cell Technology Co., Ltd. (Meisen Cell, Zhejiang, China), respectively. We verified the identity of all cell lines using Short Tandem Repeat (STR) profiling to confirm their genetic consistency. All cell lines were routinely tested for mycoplasma contamination with a commercial detection kit, and no contamination was identified throughout the experiments. For mycoplasma clearance and prevention, Mycoplasma Removal Agent (Biosharp, JP9375, Beijing, China) was added to the culture medium during routine cell maintenance. Culture media were chosen based on the specific requirements of each cell line: DMEM-F12 medium (Biosharp, BL311A) was utilized for the culture of A549 cells; while H1975, H1975 OR, PC9, and PC9 GR cells were maintained in RPMI 1640 medium (Biosharp, BL303A). To preserve the drug-resistant phenotype, osimertinib (MCE, HY-15772, Monmouth Junction, NJ, USA, 10 μM) and gefitinib (MCE, HY-50895, 0.5 μg/mL) were supplemented into the culture media of H1975 OR and PC9 GR cells, respectively. All media were fortified with 10% (v/v) South American fetal bovine serum (FBS; SinoBiological Inc., FBS05, Beijing, China), 50 U/mL penicillin, and 50 μg/mL streptomycin (Biosharp, BL505A). Cells were grown under standard conditions: 37°C, 5% CO_2_, and a humidified atmosphere.

### 2.7 Lentivirus-Mediated Gene Expression

UCK2-overexpressing (UCK2 OE) and UCK2-knockdown (shUCK2) lentiviruses were purchased from Tanjin Biotechnology Co., Ltd. (Chongqing, China). The custom vector served as the foundation for the UCK2 overexpression construct (Product No. V1004048-1) with a CMV promoter driving UCK2 expression and an EF1α promoter driving the ZsGreen-T2A-Puro selection marker. For UCK2 knockdown, the supplier provided three independent shRNA sequences in the custom vector backbone (Product No. VP013-U6-MCS-CMV-ZsGreen-PGK-Puro). Based on our pre-validation data, we selected a single, most efficient shRNA sequence (shRNA-2: GCAGGGATCTTGAGCAGATTT) for all experiments in this study, while the other two shRNA sequences (shRNA-1 and shRNA-3) were reserved for follow-up phenotypic studies in a separate manuscript. A non-targeting negative control shRNA (shNC; ACTACCGTTGTTATAGGTGT) was included to exclude off-target effects. Viral transduction was done at a multiplicity of infection (MOI) of 10 for A549 cells and 25 for H1975 cells, and 5 μg/mL polybrene (Biosharp, BL628A) was added to boost transduction efficiency. Transduced cells were selected using 2 μg/mL puromycin (Biosharp, BS111-25 mg) for 72 h to eliminate non-transduced cells and create stable cell lines. Stable cell lines were subsequently maintained in medium containing a low concentration of puromycin to preserve the transgenic phenotype. The overexpression of UCK2 was confirmed at both the mRNA and protein levels through RT-qPCR and Western blot analysis. For shUCK2, functional validation was focused on the downstream RHEB/mTOR signaling pathway (consistent with the study’s core objective to establish the UCK2-RHEB axis), and validation details for RT-qPCR and Western blot (including reference genes, antibodies, and quantification methods) are described in Section 2.8 and Section 2.9, respectively. All lentivirus-mediated experiments were independently repeated 3 times with 3 technical replicates per experiment. Cells were harvested at 48 h post-transduction for initial validation of gene overexpression or knockdown, and stable cell lines were created through puromycin selection for later long-term functional and molecular studies.

### 2.8 Isolation of RNA and Analysis via Reverse Transcription Quantitative Polymerase Chain Reaction (RT-qPCR)

**Figure A1:**
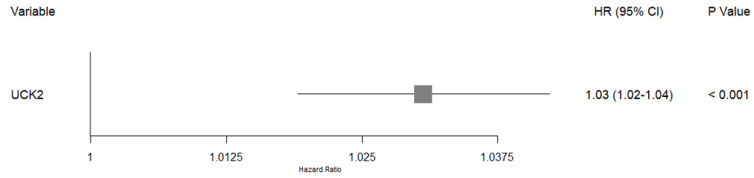
Continuous-variable Cox proportional hazards regression analysis of Uridine-cytidine kinase 2 (UCK2) expression for prognosis in lung adenocarcinoma (LUAD). Forest plot depicting the association between UCK2 mRNA expression (as a continuous variable) and overall survival (OS) in the TCGA-LUAD cohort. A hazard ratio (HR) of 1.03 (95% confidence interval [CI]: 1.02–1.04, *p* < 0.001) signifies that each unit rise in UCK2 mRNA levels corresponds to a 3% higher mortality risk, validating the consistent adverse prognostic effect of UCK2 across its entire expression range.

**Figure A2:**
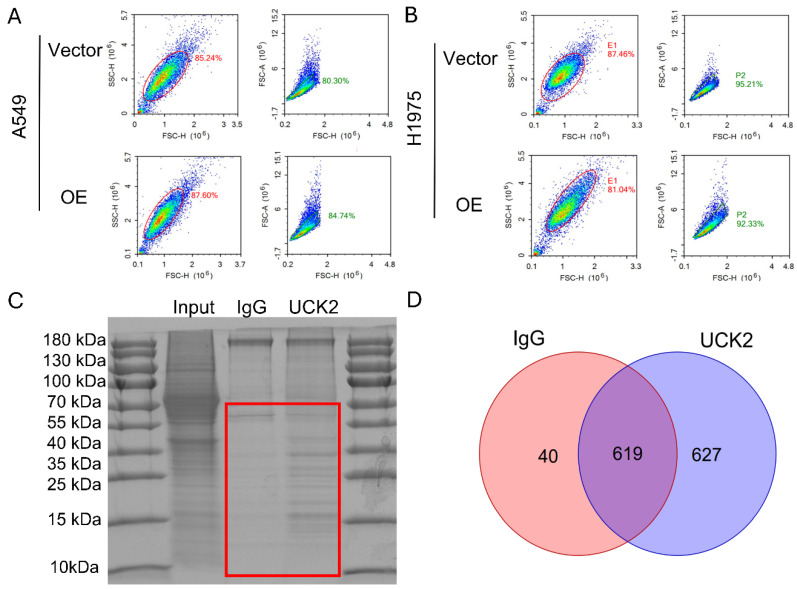
Supplementary validation for UCK2 function, IP efficiency, and interaction network. (**A**,**B**) Flow cytometry of A549 (**A**) and H1975 (**B**) cells (Vector vs. UCK2 OE). E1 (red): main cell population; P2 (green): viable cells; percentages indicate gated proportions. (**C**) Coomassie Blue-stained SDS-PAGE gel for IP efficiency validation. Red box marks the 10–55 kDa region with enriched bands in the UCK2 IP lane (vs. IgG), which were removed for LC-MS/MS. (**D**) Venn diagram of UCK2-interacting proteins found by LC-MS/MS. Numbers show unique/overlapping proteins in IgG and UCK2 IP groups.

We extracted total RNA from LUAD cells with FreeZol Reagent (Vazyme, R711, Nanjing, Jiangsu, China). RNA concentration and purity were quantified using a NanoDrop spectrophotometer. Using 500 ng of total RNA as template, we generated complementary DNA (cDNA) with the RT Master Mix for qPCR (gDNA digester plus) (MCE, HY-K0511), following the manufacturer’s protocol strictly. Quantitative PCR was performed with SYBR Green qPCR Master Mix (Universal) (MCE, HY-K0501A) on a BIOER FQD-96A Real-Time PCR System (Manufacturer: BIOER Technology Co., Ltd., Hangzhou, China). Three technical duplicates were carried out for each sample, and the reaction was conducted in a total volume of 10 μL. The thermal cycling protocol began with a 30-s denaturation step at 95°C, followed by 40 cycles that each consisting of 10 s at 95°C and 30 s at 60°C. A melt curve analysis was performed to confirm the specificity of the amplified PCR products. We normalized target gene expression against the endogenous reference gene GAPDH, and calculated relative expression levels with the 2^−ΔΔCq^ method [18]. Full primer sequences (including target genes and internal control GAPDH) are provided in Table A1. All supplementary materials for this study, and Appendix A figures (Fig. A1, Fig. A2, etc.) and Appendix A tables, are available online at the link provided in the manuscript.

### 2.9 Western Blot Analysis

Cell lysis was carried out using radioimmunoprecipitation assay (RIPA) buffer (Biosharp, BL504A), enriched with a combination of protease and phosphatase inhibitors (MCE, HY-K0010). We assessed protein concentrations using the bicinchoninic acid (BCA) assay (Epizyme Biotech, ZJ102, Shanghai, China) to guarantee consistent loading. Equal quantities of protein (20–40 μg per lane) were initially separated using sodium dodecyl sulfate-polyacrylamide gel electrophoresis (SDS-PAGE) with 10%–12.5% separating gels, and subsequently transferred to Amersham Hybond P polyvinylidene fluoride (PVDF) membranes (Cytiva, 10600023, Marlborough, MA, USA). To reduce non-specific binding, membranes were incubated with 5% non-fat milk at room temperature for 2 to 4 h, followed by an overnight incubation at 4°C with primary antibodies specific to the proteins of interest. Following extensive washes with Tris-buffered saline containing Tween-20 (TBST), membranes were incubated with species-specific horseradish peroxidase (HRP)-conjugated secondary antibodies for 1 h at room temperature. Immunoreactive protein bands were detected using an improved chemiluminescence (ECL) kit (Biocodons, ZS03033, Shanghai, China). Table A2 includes comprehensive details on all primary and secondary antibodies, such as catalog numbers, dilutions, and manufacturers.

### 2.10 RNA Sequencing and Transcriptome Profiling

For library preparation, total RNA was first isolated from harvested cell pellets (>1 × 10^6^ cells per sample) using FreeZol Reagent (Vazyme, R711). Once RNA integrity and purity were confirmed, sequencing libraries were constructed with the Illumina TruSeq Stranded mRNA Library Prep Kit (Illumina Inc., RS-122-2101, San Diego, CA, USA), strictly adhering to the standard operating guidelines provided by the manufacturer. RNA was broken down into 200–300 bp segments, and poly(A)+ mRNA enrichment was performed. These fragments were reverse transcribed to generate first- and second-strand cDNA, which was subsequently converted to double-stranded cDNA through second-strand synthesis. The resulting cDNA fragments were then processed for end repair, A-tailing, and adapter ligation. After PCR amplification, library quality was assessed using an Agilent 2100 Bioanalyzer (Agilent Technologies, Santa Clara, CA, USA) and Qubit fluorometric quantification (Qubit 4.0 Fluorometer, Q33238, Thermo Fisher Scientific, Waltham, MA, USA). Fifteen cycles of PCR amplification were carried out according to the PCR parameters outlined in the Illumina library preparation workflow. 150-bp paired-end reads were produced by sequencing the prepared libraries on an Illumina HiSeq X-ten platform (Shanghai Biotechnology Corp., Shanghai, China). For subsequent transcriptomic analysis, clean reads were aligned against the human reference genome (GRCh38) via the HISAT2 tool (version 2.2.2), and gene expression levels were measured with HTSeq-count (Shanghai Biotechnology Corp.). Differential expression analysis was conducted using the DESeq2 R package (version 1.38.0) between the UCK2 overexpression group and the vector control group (n = 3 for each group). To address the multiple testing burden inherent in gene-level analyses, the Benjamini-Hochberg procedure was utilized to perform multiple testing correction and regulate the false discovery rate (FDR). Genes with a |log_2_ FC (fold change)| ≥ 1 and an adjusted *p*-value (FDR) < 0.05 were designated as significantly differentially expressed.

### 2.11 Analysis by Co-Immunoprecipitation (Co-IP) and Subsequent Liquid Chromatography-Tandem Mass Spectrometry (LC-MS/MS)

Protein lysates from A549 cells were prepared using the lysis buffer from the immunoprecipitation (IP)/Co-IP reagent kit (MCE, HY-K0202K). For each Co-IP reaction, 1000 μg of total protein was incubated with 6 μg of anti-UCK2 antibody or an isotype-matched control IgG (Table A2) at 4°C for 12 h while continuously agitating gently. Using Protein A/G Magnetic Beads (MCE, HY-K0202K-A), immune complexes were captured for two hours at 4°C. As a loading control for Western blot analysis, 16 μg of total protein was loaded into each electrophoresis lane. The protein complexes attached to the beads were eluted and separated through SDS-PAGE. Gel slices containing the target protein bands were excised to perform in-gel proteolysis: gel pieces were destained, reduced with dithiothreitol (DTT), alkylated with iodoacetamide, and then digested overnight at 37°C using trypsin (13 ng/μL). Extracted peptides were lyophilized at −20°C under vacuum condition. LC-MS/MS assays were performed using an Orbitrap Fusion Lumos mass spectrometer (Thermo Fisher Scientific, MA, USA) integrated with an EASY-nanoLC 1200 system. An aliquot of 5 μL of the sample was injected onto a C18 column (20 cm × 75 μm i.d., 1.9 μm particle size), with separation achieved using a 60-min nonlinear gradient (4%–95% ACN, 0.1% formic acid) at a flow rate of 300 nL/min. The mass spectrometer was run in data-dependent acquisition (DDA) mode, employing higher-energy collisional dissociation (HCD) for peptide fragmentation. Full MS scans (m/z 350–1500) were obtained at a resolution of 120,000, while MS/MS scans were conducted at a resolution of 50,000, using collision energies of 25%, 30%, and 35%. Tandem mass spectra were analyzed utilizing PEAKS Studio version 13 and compared against the UniProt Homo sapiens database (2025 release) with a 1% FDR cutoff.

### 2.12 5-Ethynyl-2′-deoxyuridine (EdU) Labeling

Ninety-six well plates were prepared by seeding cells at a density of 3 × 10^3^ cells per well, utilizing cells from cultures in the logarithmic growth phase. The process involved sequentially conducting EdU labeling, fixation, permeabilization, click reaction staining, and Hoechst 33342 counterstaining, following the protocol outlined in the BeyoClick™ EdU-555 Cell Proliferation Kit (Beyotime, C0075S, Shanghai, China). The stained cells were observed and imaged using a Leica DMi8 fluorescence microscope (Leica Microsystems, Wetzlar, Germany), and ImageJ software (version 1.54d, Wayne Rasband, National Institutes of Health, Bethesda, MD, USA) was employed to quantify the EdU-positive cells.

### 2.13 Colony-Forming Assay

After infection with the indicated lentiviruses, LUAD cells were seeded into 6-well plates at a density ranging from 800 to 1000 cells per well (adjusted slightly for different cell lines to ensure comparable colony growth rates) and cultured for 14 days, with culture medium refreshed every 72 h. Upon completion of incubation, colonies were fixed in 4% paraformaldehyde for 20 min, stained with 0.5% crystal violet solution for 10 min, and then rinsed with distilled water. To evaluate colony-forming capacity, the number of colonies was manually counted using an Olympus BX53 light microscope (Olympus Corporation, BX53, Tokyo, Japan). The experiment was performed with 3 biological replicates, and each condition was set up in 3 technical replicates (wells). For statistical comparisons between the two groups, an unpaired two-tailed Student’s *t*-test was used.

### 2.14 Flow Cytometry

Cell cycle distribution and apoptotic status were assessed using the Cell Cycle and Apoptosis Analysis Kit (Beyotime, C1052), following the standard operating procedure provided by the manufacturer. To put it briefly, the cells from the UCK2 overexpressing and the empty vector groups were removed, rinsed with phosphate-buffered saline (PBS), and then incubated for 18 h at 4°C in 70% of the ice-cold ethanol. After fixation, cells were rinsed again with PBS and stained for 30 min at 37°C in the dark with propidium iodide (PI) solution containing RNase A. The final concentrations of the staining solution were: PI 50 μg/mL and RNase A 100 μg/mL, prepared according to the kit instructions (staining buffer, 20× PI stock, and 50× RNase A stock). Flow cytometry was conducted using the Agilent NovoSampler Q Flow Cytometer (Agilent Technologies, Santa Clara, CA, USA), with sample loading managed by the Agilent NovoSampler Q autosampler. Cell cycle distribution and apoptosis were analyzed using the NovoExpress software (version 1.5.0, Agilent Technologies) based on cellular DNA content and light scattering properties.

### 2.15 Transwell Assay

To evaluate cell migration and invasion capacity, A549 and H1975 cells were resuspended in serum-free medium and plated into the upper inserts of 24-well Transwell plates (8 μm pore size, LABSELECT, LABGIC, 14341, Shenzhen, China), at a density of 3 × 10^4^ cells per well, with three technical replicates per group. The lower chambers were filled with complete medium supplemented with 10% FBS to create a chemotactic gradient. In the invasion experiment, the upper inserts were pre-coated with Matrix-Gel™ Basement Membrane Matrix (Beyotime, C0371) at a 1:8 dilution ratio with serum-free medium, with 60 μL of the diluted matrix applied per insert. Inserts with the applied coating were placed in a 5% CO_2_ incubator at 37°C for a 2-h incubation period to facilitate full polymerization of the matrix. Following a 48-h incubation period under conditions of 37°C and 5% CO_2_, non-migratory cells on the upper membrane surface were removed using a sterile cotton swab. Cells that migrated to the lower membrane surface were subjected to fixation with 4% paraformaldehyde for 20 min, followed by staining with a 0.1% crystal violet solution at room temperature for 10 min. Images were captured of stained cells under an inverted light microscope, with three random fields of view selected per insert; the number of cells was quantified and analyzed via ImageJ software (version 1.54 d).

### 2.16 Wound Healing Assay

A549 and H1975 cells, following transfection, were seeded into 6-well plates at a density of 5 × 10^5^ cells per well and cultured until a confluent monolayer (≥90% confluence) was achieved. A consistent linear scratch was created across the cell monolayer with a sterile 200 μL pipette tip, and cells detached during this process were eliminated via rinsing with PBS. Each well was subsequently supplemented with serum-free medium, and the cells were cultured at 37°C for 48 h. Micrographs of the scratch site were acquired at baseline (0 h) and after 48 h of incubation using an Olympus BX53 light microscope (Olympus Corporation). The wound closure percentage was determined using the following formula: Wound closure (%) = [1 − (Wound width at 48 h/Initial wound width at 0 h)] × 100%. The experiment was performed with 3 biological replicates, and each condition was set up in 3 technical replicates (wells). Comparisons of differences between the two experimental groups were statistically evaluated via an unpaired two-tailed Student’s *t*-test.

### 2.17 Survival Analysis

The association between UCK2 expression levels and patient clinical outcomes was assessed using Kaplan–Meier survival curves, generated with the survminer R package (Version 0.4.9). The survival outcomes between patient subgroups with high and low expression of UCK2 were statistically evaluated using the log-rank test. UCK2 expression groups were defined by a median split of the expression values in the patient cohort. For multivariable analysis, Cox proportional hazards regression analysis was additionally performed to assess the independent prognostic value of UCK2 expression, adjusted for relevant clinicopathological variables.

### 2.18 Statistical Analysis

All statistical analyses were conducted using SPSS software (Version 21, SPSS Inc., Chicago, IL, USA) and GraphPad Prism 10 (GraphPad Software, San Diego, CA, USA). Between-group differences for two independent cohorts were analyzed with the Student’s *t*-test, whereas one-way analysis of variance (ANOVA) was applied to assess measurement variations among three or more independent groups. Where applicable, correlations between variables were quantified using Pearson’s correlation coefficient. All functional experiments (EdU labeling, colony formation assays, cell cycle profiling, apoptosis detection, transwell and wound healing assays) were conducted in three independent biological replicates and three technical replicates per condition to ensure reproducibility. In cases requiring multiple comparisons, statistical significance was assessed by an unpaired two-tailed Student’s *t*-test, with Bonferroni correction applied for adjustment. A *p*-value < 0.05 was defined as statistically significant.

## 3 Results

### 3.1 UCK2 Expression Profiling across Tissues and Tumors

**Figure 1:**
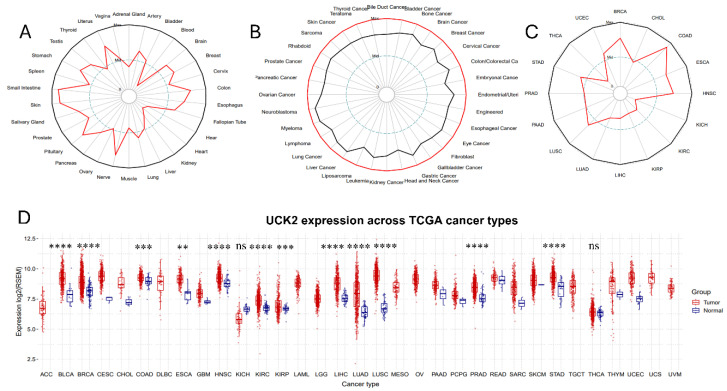
Differential expression profiling of Uridine-cytidine kinase 2 (UCK2) across normal and malignant tissues. (**A**) UCK2 mRNA expression patterns across a panel of normal human tissues. (**B**) UCK2 expression profiles in a diverse array of human tumor cell lines. (**C**) UCK2 expression in 16 types of tumors. (**D**) A comparative analysis of UCK2 expression between tumor specimens and matched normal controls. The mean ± standard deviation (SD) is used to report the data. Group differences were assessed using an unpaired two-tailed Student’s *t*-test, with significance defined as **p < 0.01, ***p < 0.001, ****p < 0.0001 (ns = not significant). Differential expression analyses were conducted only for cancer types that had ten or more paired tumor-normal samples. The standard deviation of the mean values is shown by error bars.

Analysis of the GTEx database revealed the physiological expression pattern of UCK2 across human normal tissues, with relatively high levels in the colon, nerve system, and small intestine, and lower in the lung, liver, kidney, and heart (Fig. 1A). Pan-cancer analysis using the CCLE and TCGA datasets demonstrated that UCK2 mRNA is broadly elevated across diverse cancer cell lines (Fig. 1B) and tumor tissues (Fig. 1C) compared to their normal counterparts. This tumor-associated overexpression was further corroborated by a systematic comparison of matched normal and malignant tissues in the GSCA database (Fig. 1D). Collectively, these data indicate that UCK2 is frequently overexpressed in human cancers, implicating a potential role in tumorigenesis.

### 3.2 UCK2 Is Enhanced in LUAD and Associated with Tumor Advancement

**Figure 2:**
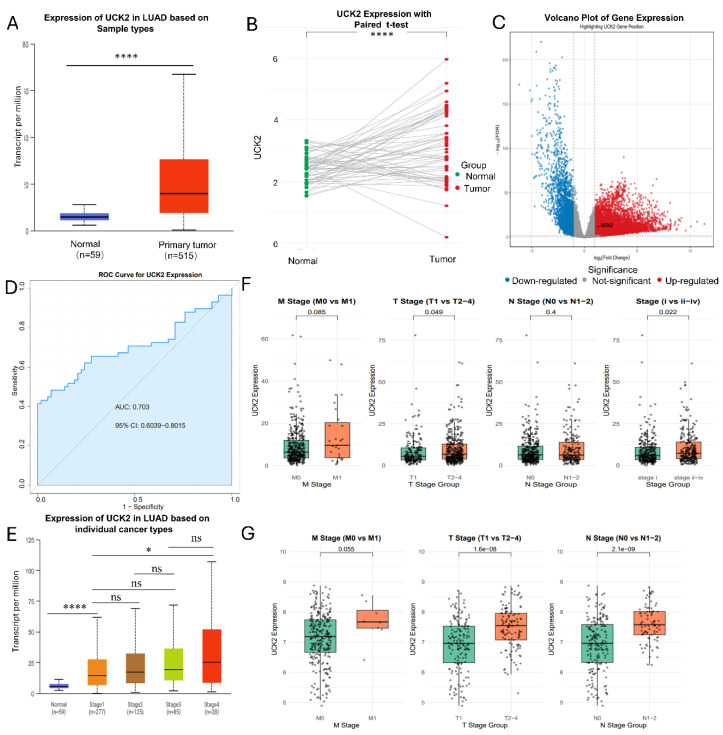
UCK2 expression is elevated in lung adenocarcinoma (LUAD) and relates to clinicopathological features (**A**) UCK2 transcript levels in LUAD tissues relative to adjacent normal controls. (**B**) UCK2 expression was analyzed in 59 paired samples of LUAD and normal tissues. (**C**) Volcano plot visualizing genome-wide differential gene expression in the TCGA-LUAD cohort. (**D**) ROC curve was used to assess how well UCK2 was used in order to distinguish between LUAD and normal tissues. (**E**) UCK2 expression levels stratified by pathological tumor stage in LUAD. (**F**) Correlation between UCK2 expression and TNM staging parameters in the TCGA-LUAD cohort. (**G**) Validation of the UCK2-TNM correlation in the independent GEO-LUAD dataset. Data in panels A, B, and E are reported as mean ± SD. Differences were analyzed using an unpaired two-tailed Student’s *t*-test (**A**), paired *t*-test (**B**), or one-way ANOVA (**E**). The standard deviation of the mean values is shown by error bars. Significance thresholds are defined as *p < 0.05, ****p < 0.0001 (ns = not significant).

Based on pan-cancer results, we focused on clarifying the clinical relevance of UCK2 in LUAD. In the TCGA-LUAD cohort, UCK2 transcript levels were substantially higher in tumor tissues relative to normal lung tissues (Fig. 2A). This upregulation was consistently verified in 59 paired LUAD tumor and adjacent normal specimens (Fig. 2B,C). Receiver operating characteristic (ROC) analysis indicated that UCK2 transcript abundance exhibits moderate diagnostic efficacy for differentiating LUAD from normal tissue, with a corresponding area under the ROC curve (AUC) of 0.703 (95% confidence interval [CI]: 0.6039–0.8015) (Fig. 2D). Furthermore, assessment of the TCGA-LUAD and GSE30219 datasets uncovered a positive association between increased UCK2 transcript levels and advanced Tumor-Node-Metastasis (TNM) stage, indicating a connection with more aggressive disease (Fig. 2E–G). In summary, UCK2 is consistently upregulated in LUAD and its transcript levels correlate with tumor progression, supporting its potential as a clinically relevant biomarker.

### 3.3 High UCK2 Expression Predicts Poor Prognosis in LUAD

**Figure 3:**
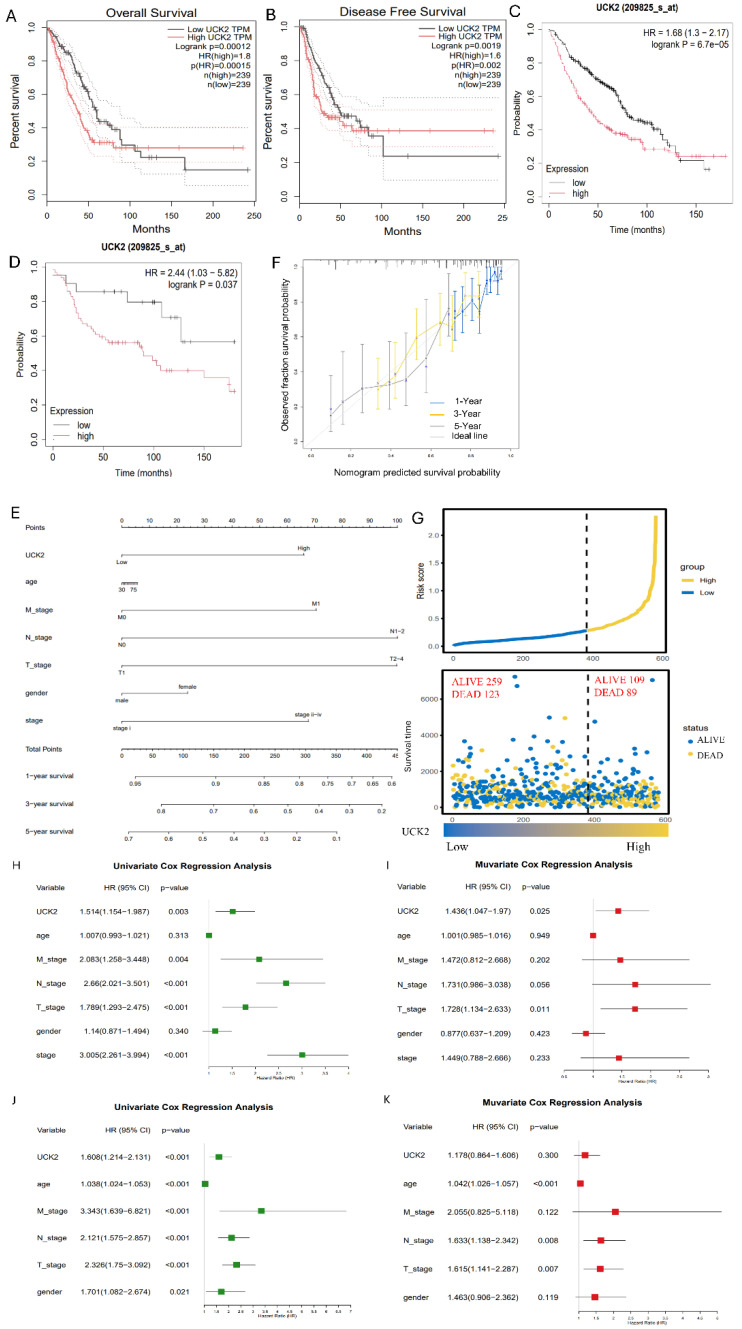
High UCK2 expression predicts unfavorable clinical outcomes in LUAD. In TCGA-LUAD patients, Kaplan–Meier survival curves for overall survival (OS, (**A**)) and disease-free survival (DFS, (**B**)) are grouped by UCK2 expression (high vs. low); OS analyses in GSE68465 (**C**) and GSE30219 (**D**) GEO cohorts (survival differences tested via Log-rank test). (**E**) Prognostic nomogram integrating UCK2 expression with clinicopathological variables (age, M/N/T-stage, gender, clinical stage) to predict 1/3/5-year OS. (**F**) Calibration curve validating the agreement between nomogram-predicted and actual observed 1-year, 3-year, and 5-year OS; curves align with the ideal line, indicating good model performance. (**G**) Risk score plot showing UCK2 expression, risk score, and survival status/time: high UCK2 expression correlates with elevated risk score and higher mortality (89 vs. 123 death cases). (**H**–**K**) Univariate and multivariate Cox proportional hazards regression analyses of UCK2’s independent prognostic value for OS: TCGA univariate (**H**), TCGA multivariate (**I**), GEO univariate (**J**), GEO multivariate (**K**). Please note that the multivariate Cox model and nomogram were generated using a TCGA subset that had complete clinical data (n = 388); a hazard ratio (HR) greater than 1 signifies an independent adverse prognostic factor.

To characterize the association between UCK2 and patient outcomes in LUAD, the TCGA-LUAD cohort was stratified into high and low UCK2 expression cohorts, with stratification based on the mean UCK2 mRNA expression level as the critical cutoff value. Kaplan–Meier survival analysis indicated that patients with high UCK2 expression experienced significantly reduced OS with a Hazard Ratio (HR) of 1.8 (*p* < 0.001) and disease-free survival (DFS) with a HR of 1.6 (*p* = 0.002) (see Fig. 3A,B). To mitigate bias from binary grouping and validate the prognostic relevance of UCK2 across its full expression spectrum, a continuous-variable Cox proportional hazards model was employed. This analysis showed that higher UCK2 expression (modeled as a continuous factor) independently predicts worse OS (HR = 1.03, 95% CI: 1.02–1.04, *p* < 0.001; Fig. A1)—confirming the consistent adverse impact of UCK2 on survival regardless of expression level. This prognostic association was further validated in two independent GEO datasets: GSE68465 (HR = 1.68, 95% CI 1.30–2.17, *p* < 0.0001) and GSE30219 (HR = 2.44, 95% CI 1.03–5.82, *p* = 0.037) (Fig. 3C,D). We built a nomogram (Fig. 3E) combining UCK2 expression and clinicopathological variables (age, M/N/T-stage, gender, clinical stage) to predict 1/3/5-year OS. The calibration curve (Fig. 3F) indicated that the predicted survival probabilities from the nomogram for 1-year, 3-year, and 5-year OS closely corresponded with the actual observed outcomes, as the curves were aligned with the ideal line, thereby confirming the model’s high predictive accuracy. A risk score plot (Fig. 3G) further showed high UCK2 expression was associated with an elevated risk score and a higher proportion of death events (89 vs. 123 cases), confirming its adverse prognostic impact. To determine whether UCK2 functions as an independent prognostic indicator, univariate and multivariate Cox proportional hazards regression analyses were performed (proportional hazards assumption verified via Schoenfeld residuals, *p* > 0.05). In the TCGA-LUAD cohort, univariate Cox analysis identified high UCK2 expression as a significant risk factor for reduced survival (HR = 1.514, 95% CI 1.154–1.987, *p* = 0.003), along with advanced T/N/M and clinical stage (Fig. 3H). After accounting for these clinicopathological variables, the multivariate analysis validated that high UCK2 expression serves as an independent predictor of unfavorable outcomes (HR = 1.436, 95% CI 1.047–1.970, *p* = 0.025) (Fig. 3I). In the GSE30219 cohort, univariate analysis also linked high UCK2 expression to shorter survival (HR = 1.608, 95% CI 1.214–2.131, *p* < 0.001) (Fig. 3J). However, this association failed to reach statistical significance in the subsequent multivariate model (HR = 1.178, 95% CI 0.864–1.606, *p* = 0.300) (Fig. 3K). Together, these findings indicate that elevated UCK2 expression consistently forecasts poor clinical outcomes in LUAD patients and serves as an independent prognostic factor within the TCGA cohort, highlighting its potential as a clinically significant biomarker.

### 3.4 UCK2 Expression Is Associated with Pro-Metastatic Pathways

**Figure 4:**
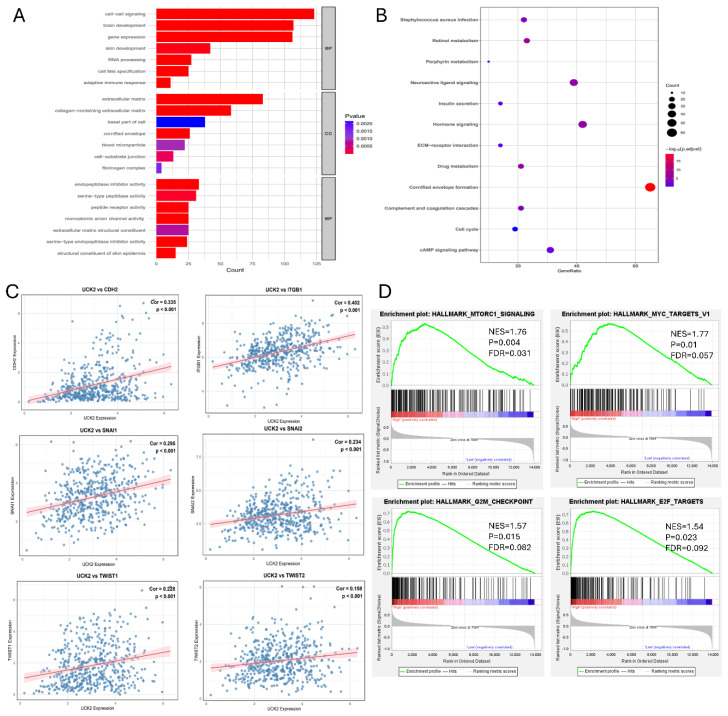
UCK2 is linked to core oncogenic signaling pathways in LUAD (**A**) GO enrichment profiling of DEGs associated with UCK2, conducted to characterize functional roles. (**B**) KEGG pathway analysis, identifying signaling cascades overrepresented in UCK2-related DEGs. (**C**) Pearson correlation analysis showing a significant link between UCK2 expression and genes involved in cancer metastasis. (**D**) GSEA plots, highlighting oncogenic signatures (MTORC1, MYC, E2F, G2M-checkpoint) associated with UCK2 expression in LUAD. Note: GO = Gene Ontology; KEGG = Kyoto Encyclopedia of Genes and Genomes; GSEA = Gene Set Enrichment Analysis; NES = Normalized Enrichment Score; FDR = False Discovery Rate.

To investigate the potential connection between UCK2 and metastasis, the TCGA cohort’s LUAD patients were split into high- and low-expression subgroups, with stratification based on the median UCK2 mRNA level as the threshold. The results of GEO and KEGG enrichment profiling were used to identify the DEGs between these groups. Results from GO profiling revealed that UCK2-high tumors displayed marked enrichment in biological processes and cellular components tied to cell-cell signaling and extracellular matrix (ECM) organization (Fig. 4A). KEGG pathway analysis also showed a significant association with ECM-receptor interaction and the cell cycle (Fig. 4B). It is noteworthy that a number of well-established pro-metastatic genes, such as Cadherin-2 (CDH2), Integrin Subunit Beta 1 (ITGB1), Snail Family Transcriptional Repressors 1 (SNAI1) and Twist Family BHLH Transcription Factors 1 (TWIST1), as well as SNAI2 and TWIST2, were positively correlated with UCK2 expression (Fig. 4C). To validate and extend these findings, GSEA was conducted using the independent GSE33532 LUAD dataset. The results confirmed significant enrichment of gene sets involved in the mTOR complex 1 (MTORC1) signaling cascade, E2 transcription factor (E2F) targets, MYC proto-oncogene (MYC) targets, and the G2/M checkpoint in samples with high UCK2 expression (Fig. 4D). Collectively, these multi-dimensional enrichment analyses indicate that elevated UCK2 expression in LUAD is closely linked to the activation of molecular networks governing metastasis, cell proliferation, and key oncogenic pathways.

### 3.5 Increased UCK2 Expression Promotes Proliferation and Advances Cell Cycle Progression in LUAD Cells

**Figure 5:**
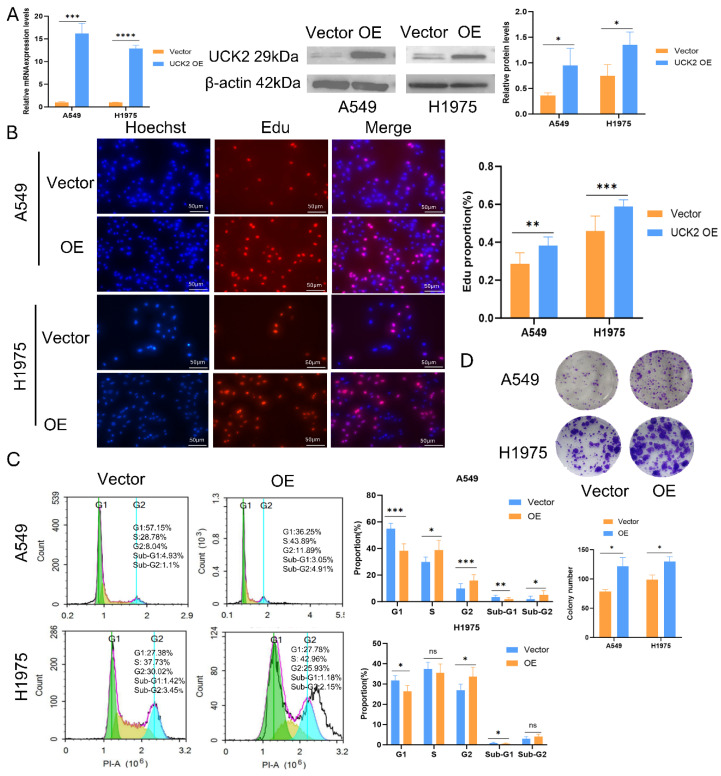
UCK2 overexpression drives LUAD cell proliferation and cell cycle progression. (**A**) Confirmation of lentivirus-induced UCK2 overexpression (OE) in A549 and H1975 LUAD cell lines using Reverse Transcription Quantitative Polymerase Chain Reaction (RT-qPCR) and Western blotting. (**B**) EdU incorporation assay showing that UCK2 OE markedly boosts DNA biosynthesis and proliferation in A549 and H1975 cells. Hoechst (blue) stains cell nuclei, while EdU (red) indicates newly synthesized DNA; scale bar = 50 μm. (**C**) Analysis of cell cycle distribution in A549 and H1975 cells using flow cytometry following UCK2 OE. (**D**) Colony formation assay revealing increased clonogenic capacity with UCK2 OE. Note: Error bars indicate the SD of the mean. Data are reported as mean ± SD from independent experiments. Group differences were evaluated using an unpaired two-tailed Student’s *t*-test (*p < 0.05, **p < 0.01, ***p < 0.001, ****p < 0.0001; ns = not significant).

In order to functionally characterize the role of UCK2 in LUAD, stable UCK2-overexpressing models were created in A549 and H1975 cells, with the successful overexpression of UCK2 confirmed at both the mRNA and protein levels (Fig. 5A). EdU incorporation experiments demonstrated that UCK2 overexpression considerably boosted the proliferative capacity of both A549 and H1975 cell lines (Fig. 5B). This pro-proliferative effect was further validated by colony formation assays, which found a notable increase in clonogenic capacity after UCK2 overexpression (Fig. 5D). Consistently, UCK2 overexpression accelerated cell cycle progression and modestly suppressed apoptosis in both A549 and H1975 cells. However, phase-specific changes varied by cell line: in A549 cells, UCK2 overexpression led to significant G1-phase reduction alongside increases in S-phase and G2-phase; in H1975 cells, by contrast, significant shifts were limited to G1-phase decrease and G2-phase increase, with no notable change in S-phase (Fig. 5C and Fig. A2A,B). Together, these functional assays demonstrate that forced UCK2 expression drives cell cycle progression, inhibits apoptosis, and ultimately enhances the proliferative and clonogenic potential of LUAD cells *in vitro*.

### 3.6 UCK2 Overexpression Promotes Motility of LUAD Cells

**Figure 6:**
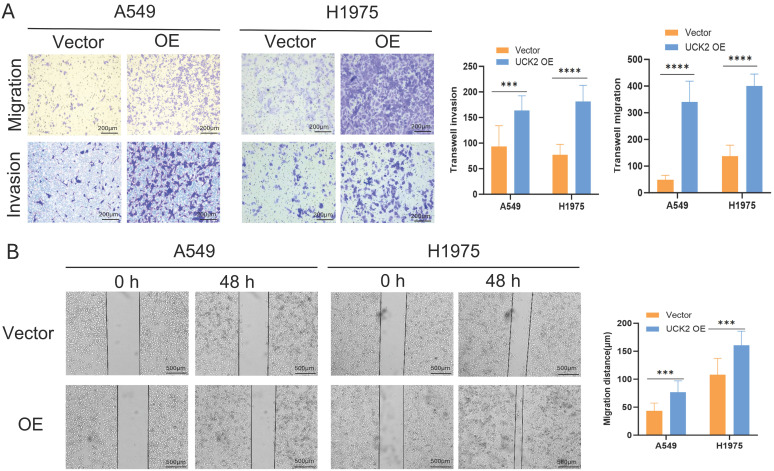
UCK2 overexpression (OE) increases the migratory and invasive phenotypes of LUAD cells. (**A**) Transwell migration and invasion assays, which assessed the motility of A549 and H1975 cells in response to UCK2 OE. Scale bar = 200 μm. (**B**) Wound-healing assay, used to evaluate the *in vitro* migration potential of UCK2 OE A549 and H1975 cells. Scale bar = 500 μm. Note: Error bars indicate the SD of the mean. Data are reported as mean ± SD from independent experiments. An unpaired two-tailed Student’s *t*-test was used to examine group differences (***p < 0.001, ****p < 0.0001).

To investigate how UCK2 affects LUAD cell motility, transwell migration and invasion assays were used to examine the effects of UCK2 overexpression. Results demonstrated that UCK2 overexpression led to considerable increases in both migratory and invasive capacities of A549 and H1975 cells (Fig. 6A). These findings were supported by the results of wound healing assays: after scratching confluent monolayers of A549 or H1975 cells, UCK2 overexpression significantly accelerated lateral migration into the wound area within 48 h (Fig. 6B). In summary, these findings confirm that UCK2 serves as a key regulator of cell motility, as reflected by the robust enhancement in migratory capacity following UCK2 overexpression.

### 3.7 UCK2 Methylation, Expression, and Clinical Outcome in LUAD

**Figure 7:**
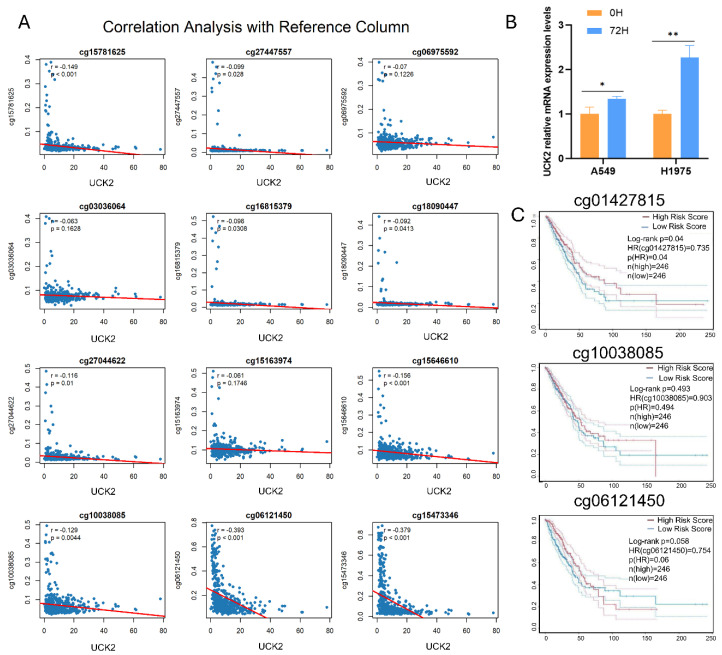
UCK2 promoter methylation modulates its expression and prognostic impact in LUAD. (**A**) Scatter plots depicting the relation between UCK2 mRNA expression and methylation status at individual CpG sites, analyzed by Pearson correlation, with correlation coefficients and statistical significance reported. (**B**) Alterations in UCK2 expression following 72 h of decitabine treatment. (**C**) Kaplan-Meier survival curves for correlation analysis between methylation levels of specific CpG sites (cg01427815, cg10038085, cg06121450) and LUAD prognosis, assessed by the Log-rank test. Note: Error bars indicate the SD of the mean. Statistical significance is denoted as follows: *p < 0.05, **p < 0.01.

**Figure A3:**
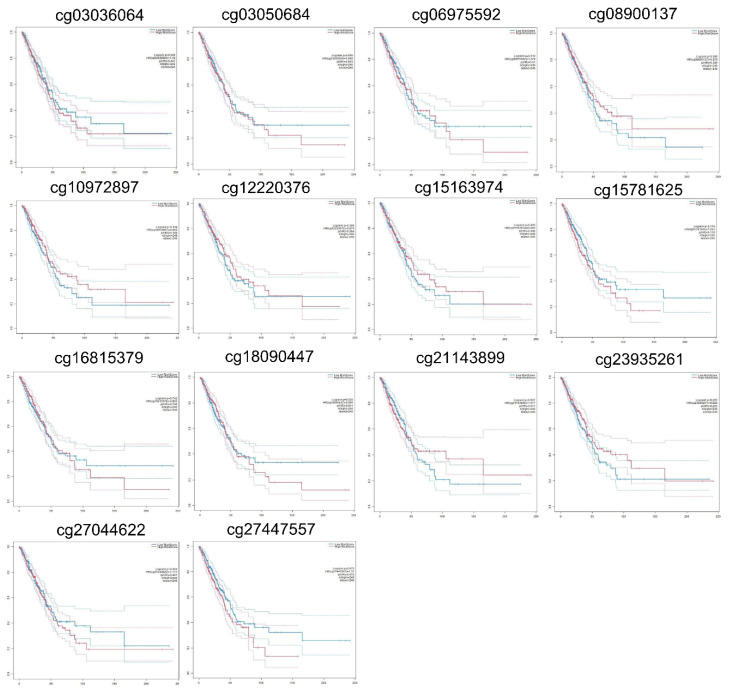
Survival analysis of UCK2 methylation at additional CpG sites in LUAD. Kaplan-Meier curves depict the relationship between methylation levels and overall survival for all other CpG sites analyzed, which exhibited no statistically significant association with prognosis (log-rank test, all *p* > 0.05).

The analysis of the TCGA-LUAD data suggested decreased methylation at the UCK2 locus. To investigate this in more detail, we analyzed 12 key cytosine-phosphate-guanine (CpG) sites located within the UCK2 gene. Spearman correlation analysis revealed an overall inverse relationship between UCK2 mRNA levels and DNA methylation, with the strongest negative correlations observed at sites cg06121450 and cg15473346 (Fig. 7A). In line with this, the treatment of A549 and H1975 LUAD cells with Decitabine, a DNA methyltransferase inhibitor at a concentration of 10 μM, led to a significant increase in UCK2 expression (Fig. 7B). After that, we assessed the prognostic value of UCK2 methylation. Patient stratification based on specific CpG sites revealed that methylation at cg01427815 was significantly associated with patient outcome (HR = 0.735, *p* = 0.04). This significant site, along with two representative non-significant sites, is presented in Fig. 7C. Survival curves for all other analyzed CpG sites that showed no substantial correlation are provided in Fig. A3. In conclusion, while UCK2 expression is epigenetically regulated by promoter methylation—a finding validated *in vitro*—the methylation status at individual CpG sites, with the exception of cg01427815, does not serve as a robust independent prognostic indicator in this LUAD cohort.

### 3.8 UCK2 Expression Is Associated with Aberrant Immune Cell Infiltration in LUAD

**Figure 8:**
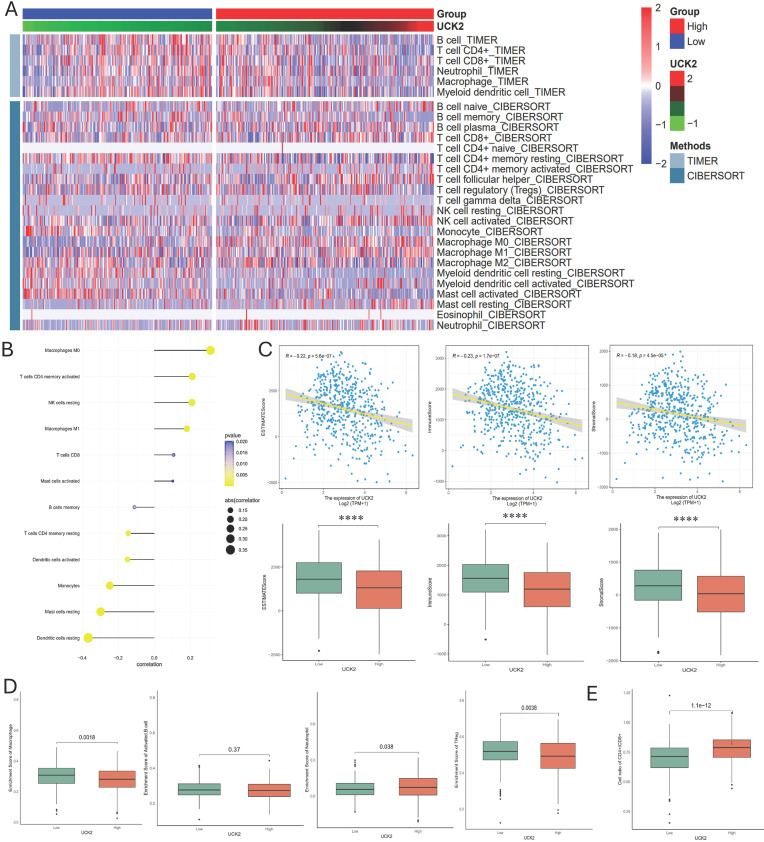
UCK2 expression and its link to immune cell infiltration in LUAD. (**A**) Heatmap illustrating differential immune cell infiltration levels in UCK2 high- and low-expression LUAD patients, evaluated via TIMER and CIBERSORT. (**B**) Lollipop plot depicting the correlation of UCK2 expression with immune cell subsets. (**C**) ESTIMATE-based analysis correlating UCK2 expression with immune/stromal scores across patient groups. (**D**) Comparative analysis of regulatory macrophages, activated B cells, neutrophils, and Treg cells across UCK2 high/low-expression patient groups. (**E**) Differential analysis of the CD4^+^/CD8^+^ cell ratio between UCK2 high/low-expression patients. Note: Statistical significance was determined by unpaired two-tailed Student’s *t*-test. Error bars indicate mean ± SD. ****p < 0.0001. ES, Enrichment Score.

**Figure A4:**
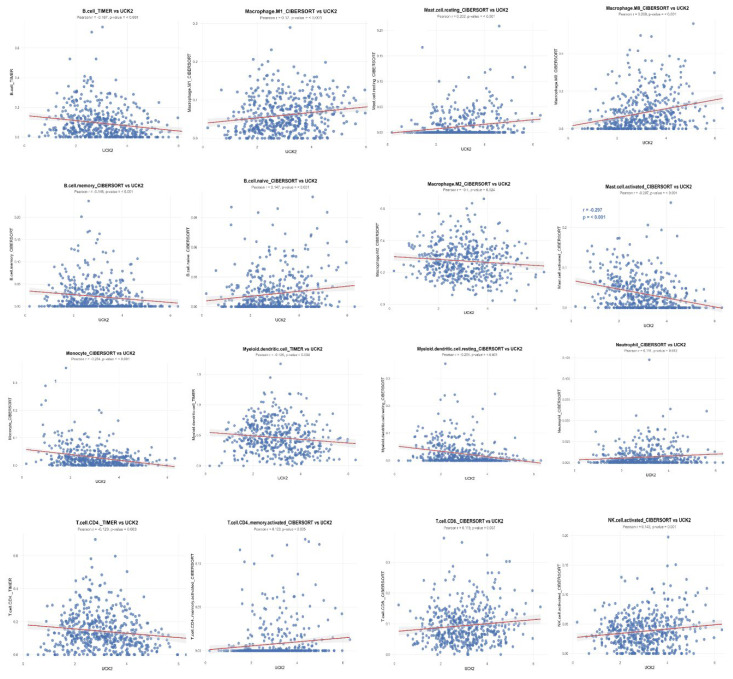
Correlation scatter plots of UCK2 expression with tumor infiltration levels of individual immune cell subsets in LUAD. Scatter plots depict the relationship between UCK2 mRNA expression (*x*-axis) and the estimated infiltration level (*y*-axis) for each immune cell type analyzed via CIBERSORT/TIMER.

To characterize the crosstalk between UCK2 and the tumor immune microenvironment (TME), multiple computational approaches were implemented. CIBERSORT and TIMER analyses showed that increased UCK2 expression was associated with advanced infiltration of specific immune cell subsets, including M0 macrophages (non-polarized), M1 macrophages (classically activated), CD4^+^ memory T cells, CD8^+^ T cells, and natural killer (NK) cells, but reduced levels of resting dendritic cells, mast cells, monocytes, and B cells. This distinct infiltration pattern is summarized in a heatmap (Fig. 8A), with correlative relationships visualized in a lollipop plot (Fig. 8B) along with detailed scatter plots for each subset of immune cell (Fig. A4). This association, however, was not uniformly reflected by a global microenvironment score. Application of the ESTIMATE algorithm, which calculates comprehensive immune and stromal scores, showed an inverse correlation between UCK2 expression and both scores (Fig. 8C). This noticeable discrepancy probably arises from the different analytical scopes, whereas CIBERSORT and TIMER focus on specific cell types, ESTIMATE evaluates the overall immune and stromal components. Further analysis using ssGSEA indicated that high UCK2 levels related to decreased infiltration of macrophages and regulatory T (Treg) cells, but increased neutrophil infiltration (Fig. 8D). Additionally, a higher CD4^+^/CD8^+^ T cell ratio was observed in the high UCK2 expression group (Fig. 8E). This distinct pattern of immune subset redistribution, rather than a uniform change in overall infiltration, defines the immune-dysfunctional landscape associated with high UCK2 expression in LUAD.

### 3.9 UCK2 Expression Is Associated with Altered Therapeutic Response in LUAD

**Figure 9:**
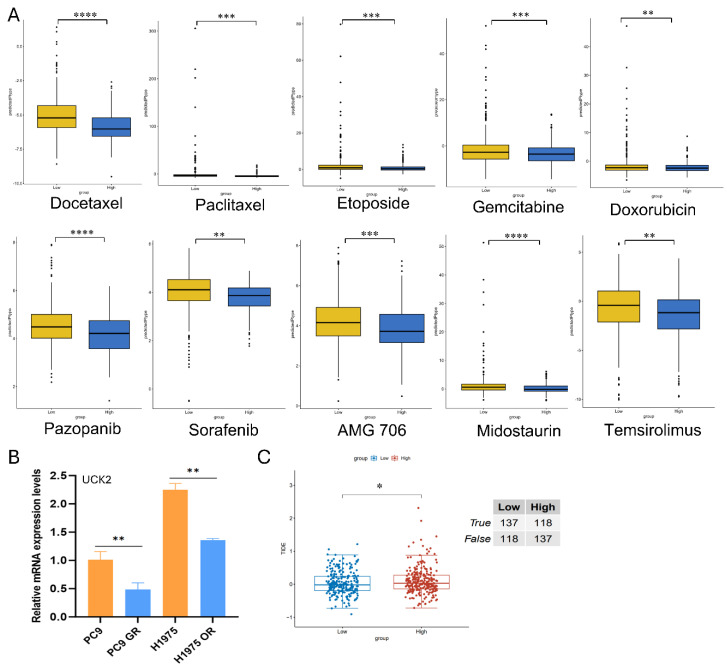
Correlation between UCK2 expression and both chemoresistance and response to immunotherapy in LUAD. (**A**) Box plots showing estimated IC_50_ values of five chemotherapeutic and five targeted agents in patients with high versus low UCK2 expression. (**B**) Expression of UCK2 in TKI-resistant cell lines. (**C**) Box plots showing differences in immunotherapy responses between patients with high and low UCK2 levels, as well as Tumor Immune Dysfunction and Exclusion (TIDE) scores. The mean ± SD of the data is shown (the same is represented by error bars). Statistical significance was tested by unpaired two-tailed Student’s *t*-test. *p < 0.05, **p < 0.01, ***p < 0.001, ****p < 0.0001.

We employed *in silico* strategies to explore potential links between UCK2 expression and drug sensitivity. Analysis using the pRRophetic algorithm indicated that elevated UCK2 expression may correlate with markedly reduced predicted IC_50_ values for multiple agents, including docetaxel and paclitaxel (Fig. 9A). Intriguingly, in experimental models of acquired resistance to tyrosine kinase inhibitors (TKIs), UCK2 expression was downregulated in PC9 GR and H1975 OR cells (Fig. 9B). Next, we employed the TIDE algorithm to evaluate the potential for tumor immune evasion; patients with high UCK2 expression showed increased TIDE scores (Fig. 9C). According to the algorithm, a higher TIDE score indicates a greater predicted probability of primary resistance to ICIs directed against programmed cell death protein 1 (PD-1) and cytotoxic T-lymphocyte-associated protein 4 (CTLA-4). Collectively, these *in silico* and experimental observations point to a complex, context-dependent role for UCK2 in therapeutic response in LUAD. While high expression showed associations with sensitivity to certain agents and a higher predicted risk of ICI non-response, its expression was suppressed in models of acquired TKI resistance.

### 3.10 UCK2 Promotes LUAD Progression by Engaging the RHEB/mTOR Signaling Axis

**Figure 10:**
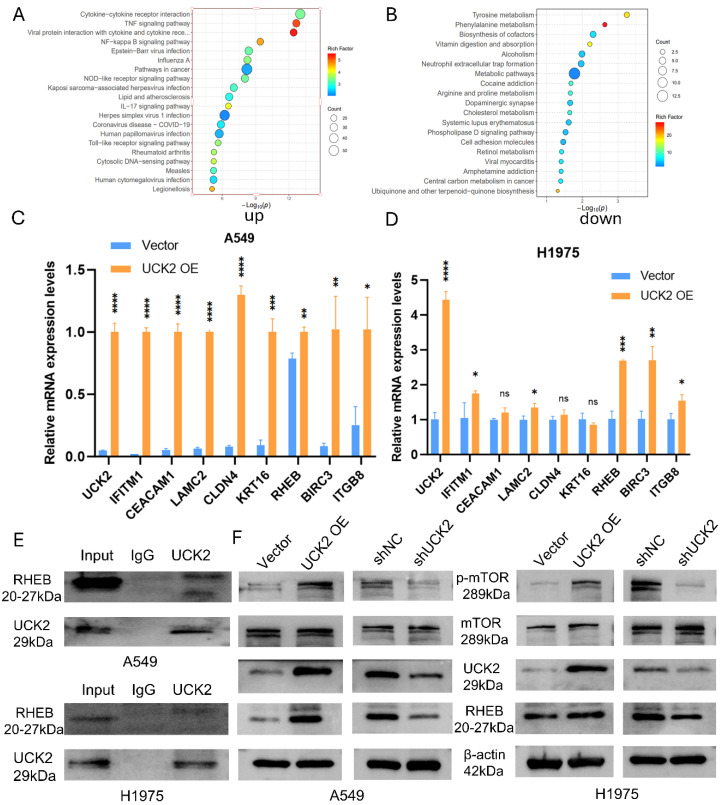
UCK2 activates the RHEB/mTOR signaling pathway to promote LUAD progression. (**A**,**B**) GSEA profiling of RNA-seq datasets from UCK2-overexpressing A549 cells revealed significantly enriched pro-tumorigenic pathways and suppressed metabolic pathways. (**C**,**D**) Validation of representative differentially expressed genes (DEGs) by RT-qPCR in A549 and H1975 cells with UCK2 overexpression; RHEB mRNA level was considerably elevated along with pro-tumorigenic genes ITGB8 and BIRC3. (**E**) Co-immunoprecipitation (Co-IP) assay shows endogenous RHEB was found in UCK2 immunoprecipitates (IgG as negative control), supporting a physical link between UCK2 and RHEB in LUAD cells. (**F**) Western blot analysis shows UCK2 overexpression elevates protein levels of RHEB and phosphorylated mTOR (p-mTOR), while UCK2 knockdown reduces RHEB and p-mTOR levels; total mTOR protein level remains unchanged in both models, confirming UCK2 positively regulates the RHEB/mTOR signaling axis. *p < 0.05, **p < 0.01, ***p < 0.001, ****p < 0.0001, ns = not significant.

To dissect the oncogenic mechanisms driving UCK2 function in LUAD, we performed RNA-seq in A549 cells stably overexpressing UCK2. GSEA revealed that UCK2 overexpression significantly enriched transcripts linked to pro-tumorigenic signaling pathways—encompassing tumor necrosis factor (TNF) and nuclear factor-κB (NF-κB) signaling cascades—while downregulating genes involved in metabolic processes (Fig. 10A,B). To validate these transcriptomic findings, we quantified mRNA levels of representative DEGs linked to tumor progression. The overexpression of UCK2 significantly increased mRNA expression of the small GTPase Ras homolog enriched in brain (RHEB) in both A549 and H1975 cells, as well as other pro-tumorigenic transcripts such as integrin subunit beta 8 (ITGB8) and BIRC3 (baculoviral IAP repeat-containing protein 3) (Fig. 10C,D).

To identify molecular interactors of UCK2, we conducted Co-IP coupled with LC-MS/MS in A549 cells. The efficiency of IP was confirmed by Coomassie Blue staining performed on SDS-PAGE gels; the 10–55 kDa region, which showed visibly enriched protein bands in the UCK2 IP lane relative to the IgG control, was excised for LC-MS/MS analysis (Fig. A2C). This approach identified 627 proteins that specifically co-precipitated with UCK2 (Fig. A2D). Among these high-confidence interactors, we prioritized RHEB—a well-characterized upstream activator of MTORC1 [19,20]—for further mechanistic evaluation. Using a specific anti-UCK2 antibody for immunoprecipitation, independent Co-IP assays demonstrated that endogenous RHEB was detected in UCK2 immunoprecipitates, whereas no RHEB signal was observed in the parallel IgG negative control group, confirming a physical association between UCK2 and RHEB in LUAD cells (Fig. 10E). At the protein level, UCK2 overexpression in A549 cells increased RHEB abundance and enhanced phosphorylation of mTOR at Ser2448 (p-mTOR Ser2448), with no detectable change in total mTOR levels. Conversely, UCK2 knockdown (shUCK2) in both A549 and H1975 cells reduced RHEB protein levels and blunted mTOR phosphorylation, while total mTOR expression remained unchanged (Fig. 10F).

## 4 Discussion

LUAD continues to pose a daunting clinical challenge. This is due to its strong tendency for metastasis [21,22] and the common development of resistance to multiple therapies, including chemotherapy [23], targeted agents [7,24], and immunotherapy [24,25]. In this context, our integrated multi-omics analysis identifies UCK2 as a key oncogenic driver in LUAD. We demonstrate that UCK2 is markedly upregulated in LUAD specimens and serves as a promising independent prognostic factor for poor clinical outcomes. This clinical correlation is functionally grounded: UCK2 overexpression potently enhances LUAD cell proliferation, cell cycle progression, and migration *in vitro*, while concurrently increasing RHEB protein levels and phospho-mTOR (p-mTOR) activation; conversely, knockdown of endogenous UCK2 reduces RHEB abundance and impairs mTOR pathway activity (Fig. 10F). These gain- and loss-of-function data directly validate UCK2’s dual role in promoting oncogenic phenotypes and regulating the RHEB/mTOR axis, firmly establishing UCK2 as a bona fide driver of LUAD aggressiveness—transcending its role from a passive correlative marker to a promising candidate for both prognostic stratification and therapeutic intervention. Notably, while UCK2 itself has been externally validated, the integrated prognostic nomogram combining UCK2 with clinical variables awaits future validation in independent prospective cohorts to confirm its clinical utility.

The epigenetic regulation underlying UCK2 overexpression offers a novel mechanistic insight into its upregulation in lung adenocarcinoma. Our data reveal that high UCK2 expression in LUAD is inversely associated with DNA methylation levels at specific promoter CpG sites (e.g., cg06121450), a regulatory relationship functionally validated by decitabine treatment *in vitro*. This finding positions DNA hypomethylation as a key permissive mechanism enabling high UCK2 transcription in tumors. Interestingly, the prognostic relevance of this methylation appeared limited to a single CpG site (cg01427815), suggesting that while epigenetic dysregulation initiates high UCK2 expression, the ensuing aggressive phenotype and patient outcome are likely dominated by the downstream molecular functions of UCK2 itself.

Beyond the established roles of UCK2 in the PI3K/AKT/mTOR network [10] and its epigenetic regulation [11], emerging evidence suggests additional oncogenic mechanisms—such as non-coding RNA-mediated modulation of EGFR signaling [13], metabolic stress-induced regulation of UCK2 stability [26], and recent identification of UCK2 turnover control by the CTLH-WDR26 E3 ligase [27]—highlighting its functional complexity and context-dependent oncogenicity. Extending this understanding, our work identifies a novel molecular link in LUAD: UCK2 forms a specific biochemical association with the small GTPase RHEB, a critical activator of MTORC1 [19,20] (validated by Co-IP, Fig. 10E). While prior studies have implicated UCK2 in the broader PI3K/AKT/mTOR network (e.g., promoting cisplatin resistance in intrahepatic cholangiocarcinoma [10] or reported a direct UCK2-mTOR interaction in other contexts [12], our findings define a more precise mechanism in LUAD: UCK2 engages this central oncogenic pathway through RHEB. Specifically, changes in UCK2 expression correlate with RHEB protein abundance and p-mTOR levels, positioning the UCK2-RHEB module as an upstream regulator of mTOR pathway activity. This model is strongly supported by multiple lines of evidence: transcriptional profiles of high-UCK2 LUAD cells show enrichment of E2F and MYC targets (canonical downstream effectors of mTOR signaling [25,28]); UCK2 expression exhibited a positive correlation with transcription factors implicated in EMT (Fig. 4C); and functional assays confirm enhanced migratory and invasive capacities in UCK2-overexpressing cells—consistent with mTOR’s well-documented role in mediating pro-metastatic behaviors. Collectively, our molecular and functional data converge to support a model wherein UCK2 promotes LUAD progression, at least in part, by forming a functional complex with RHEB to activate mTOR signaling. Notably, mTOR signaling is well recognized to bridge tumor-intrinsic oncogenic pathways with TME remodeling, particularly immune modulation [29]—prompting us to explore whether UCK2 might also shape the immune landscape of LUAD.

Immunotherapy (notably immune checkpoint blockade, ICB) has transformed cancer therapy, yet its efficacy is constrained by primary or acquired resistance, often driven by oncogenic signaling and immune dysfunction [30]. Our multi-algorithm analysis (CIBERSORT, ssGSEA, ESTIMATE, TIDE) reveals that high UCK2 expression correlates with an immune-dysfunctional landscape: altered myeloid cell infiltration, elevated CD4^+^/CD8^+^ T-cell ratio, reduced overall immune and stromal content, and increased immune evasion potential—signatures linked to poor ICB response [31,32,33,34]. Mechanistically, this connection may be mediated by UCK2-driven mTOR activation, as mTOR signaling modulates immune cell activity and fosters immune dysfunction within tumors [29]. Importantly, our data establish a correlation, not causation, between the UCK2-RHEB-mTOR axis and immune evasion—whether UCK2 directly bridges tumor-intrinsic signaling and immune modulation remains a testable hypothesis for future work.

The relationship between UCK2 and therapeutic response presents a complex, context-dependent picture that warrants careful consideration. Our pharmacogenomic analysis, based on computational predictions, indicated a potential association with increased sensitivity to a broad spectrum of agents in high-UCK2 tumors, supporting its exploratory potential as a candidate predictive biomarker. Specifically, analysis using the pRRophetic algorithm associated high UCK2 expression with lower predicted IC_50_ for taxanes and mTOR pathway inhibitors (Fig. 9A). This observation is biologically plausible, as UCK2-driven mTOR activation may render high-UCK2 tumors more dependent on mTOR signaling, thereby increasing sensitivity to mTOR inhibitors [35]. These bioinformatic observations propose a hypothesis: UCK2 might serve as a candidate biomarker, warranting future investigation into whether it can identify patients more likely to benefit from regimens containing taxanes or mTOR inhibitors like Everolimus [35]. Thus, these predictive data generate testable hypotheses for future biomarker-driven research. Notably, the observed downregulation of UCK2 in models of acquired resistance to EGFR TKIs introduces a crucial nuance. This apparent paradox mirrors context-specific findings in other malignancies, such as the association of UCK2 with cisplatin resistance in cholangiocarcinoma [10], and underscores that the functional consequence of UCK2 expression may be tightly linked to specific therapeutic pressures. In summary, the context-dependent associations of UCK2 with drug response underscore that any potential application in treatment stratification would require rigorous validation and a highly personalized assessment.

In evaluating UCK2’s druggability, we identified small-molecule inhibitors reported in PubChem (AID: 1589135) and ChEMBL (ID: CHEMBL4387680) [36], confirming UCK2 as a pharmacologically tractable target. Additionally, mTOR (the downstream effector of the UCK2-RHEB axis) is clinically validated with approved inhibitors [35,37], de-risking translational efforts by targeting a well-characterized pathway downstream of UCK2. Future directions include validating UCK2-mTOR inhibitor combinations in immunocompetent animal models and leveraging structure-based design (e.g., AlphaFold for UCK2 structure, UniProt: Q9BZX2) to develop selective inhibitors.

Finally, the study has several defined boundaries that point to productive future directions. A primary methodological limitation is the reliance on gain-of-function (overexpression) approaches to define UCK2’s oncogenic potential. While the *in vitro* and bioinformatic evidence presented is robust, the functional validation of the UCK2-RHEB-mTOR axis, particularly its role in metastasis and immune modulation, awaits confirmation in immunocompetent animal models—a key objective of our ongoing research. The primary aim here was to establish the novel UCK2-RHEB connection and its pathological relevance; a systematic interrogation of the phenotypic consequences of endogenous UCK2 loss across all cancer hallmarks is the focus of a subsequent, dedicated study. Furthermore, beyond the external validation of UCK2 as a biomarker, the clinical predictive power of the combined prognostic model (nomogram) requires assessment in prospective cohorts. Finally, dissecting how UCK2 coordinates its metabolic and signaling roles presents an intriguing mechanistic avenue for future work.

## 5 Conclusions

In summary, this integrated multi-omics and functional analysis establishes UCK2 as a pivotal driver of LUAD pathogenesis. We demonstrate that UCK2 is epigenetically dysregulated (via promoter hypomethylation), associates with and regulates RHEB to activate mTOR signaling, and its expression correlates with an immune-dysfunctional TME—with key findings validated by RT-qPCR, Co-IP, and loss-of-function experiments. These results solidify UCK2 as a candidate prognostic biomarker (externally validated in GEO cohorts) and a therapeutic target that bridges growth signaling and immune evasion. Future translation should focus on three priorities: (1) evaluating existing UCK2 inhibitors in the context of the UCK2-RHEB-mTOR axis; (2) validating the clinical nomogram in independent prospective cohorts; and (3) exploring UCK2’s utility in stratifying patients for combination therapies (e.g., mTOR inhibitors, ICB)—with the ultimate goal of improving personalized treatment outcomes for LUAD patients.

## Data Availability

The transcriptomic and clinical datasets used in this study are freely accessible. TCGA-LUAD data is available through the Genomic Data Commons (GDC) portal, and GEO datasets (GSE30219, GSE68465) can be obtained from the Gene Expression Omnibus (GEO) website. Data generated in this study have been deposited in the following repositories: RNA-seq data (UCK2 overexpression in A549 cells): NCBI Sequence Read Archive (SRA) under BioProject accession [PRJNA1398051] (SRA submission ID: SUB15914467), held in private access until publication. Mass spectrometry proteomics data: ProteomeXchange Consortium via iProX, with dataset identifier [PXD073030]. All additional datasets that support the findings presented in this study can be obtained from the corresponding author upon reasonable request.

## Supplementary Materials

The supplementary material is available online at https://www.techscience.com/doi/10.32604/or.2026.078651/s1.

## Appendix A

**Table A1:** Primers for RT-qPCR.

Gene Name	Primer Sequences
GAPDH	F108: 5′-GGAGCGAGATCCCTCCAAAAT-3′ R304: 5′-GGCTGTTGTCATACTTCTCATGG-3′
UCK2	F57: 5′-GCCCTTCCTTATAGGCGTCAG-3′ R162: 5′-CTTCTGGCGATAGTCCACCTC-3′
RHEB	F92: 5′-TTGTGGACTCCTACGATCCAA-3′ R186: 5′-GGCTGTGTCTACAAGTTGAAGAT-3′

**Table A2:** Antibodies utilized in this study.

Protein Name	Manufacture (Catalog Number)	Experimental Use (Working Dilution)	Manufacturer Website
β-actin	HUABIO (Hangzhou, Zhejiang, China) (HA722023)	WB (1:15000)	https://huabio.cn/products/beta-Actin-antibody-HA722023
UCK2	Abcam (Cambridge, UK) (AB241281)	WB (1:1000) IP (1:150)	https://www.abcam.cn/products/primary-antibodies/uck2-antibody-ab241281.html
p-mTOR	Sellect (Houston, TX, USA) (F2584)	WB (1:1000)	https://www.selleck.cn/antibodies/phospho-mtor-ser2448-antibody-p18p17.html
mTOR	Sellect (Houston, TX, USA) (F0169)	WB (1:1000)	https://www.selleck.cn/antibodies/mtor-antibody-c15m14.html
RHEB	MCE (MedChemExpress, Monmouth Junction, NJ, USA) (HY-P80310)	WB (1:1000)	https://www.medchemexpress.cn/antibody/rheb-rabbit-mab.html
Rabbit IgG Isotype Control	MCE (MedChemExpress, Monmouth Junction, NJ, USA) (HY-P80879)	IP (1:20)	https://www.medchemexpress.cn/antibody/rabbit-igg-5b2-mouse-mab.html
antiRabbit secondary antibody	Abgent (San Diego, CA, USA) (ASS1009)	WB (1:5000)	http://www.abgent.com/products/ASS1009-Goat-Anti-Rabbit-IgGHL-MouseHuman-ads-HRP-Secondary-Antibody

Note: WB (Western blotting); IP (Immunoprecipitation).
