# Supplementary material for: UCK2 Drives Lung Adenocarcinoma Progression and Immune Dysregulation via the RHEB/mTOR Signaling Axis
Source: Oncol Res. 2026 May 21;34(6):26. doi: 10.32604/or.2026.078651 (PMC13223187; doi:10.32604/or.2026.078651)
Supplement: Supplementary file 1 [file OncolRes-34-78651-s001.zip › TSP_OR_78651-s001.docx]

**Supplementary Uncropped Western Blot Legends**

**1.File Name: Supplementary_WB_Uncropped_Fig5A.tiff**

Corresponding Main Figure: Fig. 5A

Experiment: UCK2 overexpression validation in A549 and H1975 cells

Lane Annotation: Protein Marker (10-180 kDa) → A549/H1975-Vector → A549/H1975-UCK2-OE (Core bands marked with red rectangles, corresponding to the cropped image in the main text)


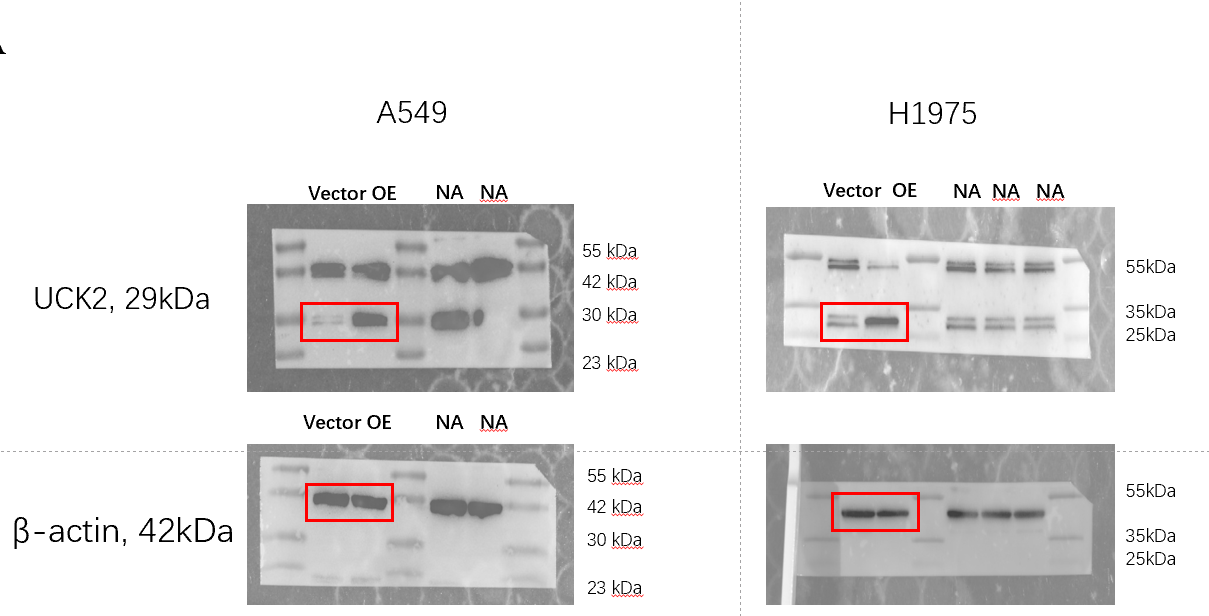


**2. File Name: Supplementary_WB_Uncropped_Fig10E.tiff**

Corresponding Main Figure: Fig. 10E

Experiment: UCK2-RHEB Co-immunoprecipitation (Co-IP) in A549 cells

Lane Annotation: Protein Marker (10-180 kDa) → Input → IgG (negative control) → UCK2-IP (Core bands marked with red rectangles, corresponding to the cropped image in the main text)
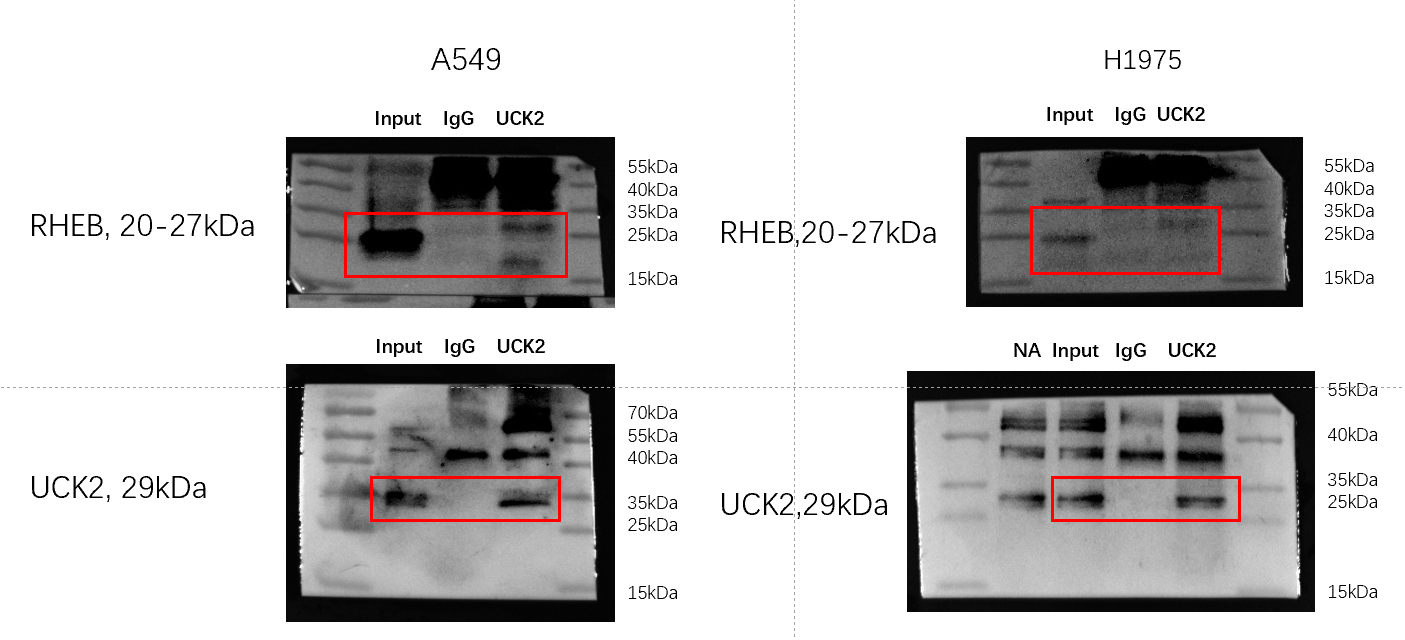


**3. File Name: Supplementary_WB_Uncropped_Fig10F.tiff**

Corresponding Main Figure: Fig. 10F

Experiment: RHEB/p-mTOR expression detection in A549/H1975 cells (UCK2 OE/knockdown)

Lane Annotation: Protein Marker (10-250 kDa) → A549/H1975-Vector → A549/H1975-UCK2-OE → Protein Marker →A549/H1975-shNC → A549/H1975-shUCK2 (Core bands marked with red rectangles, corresponding to the cropped image in the main text)
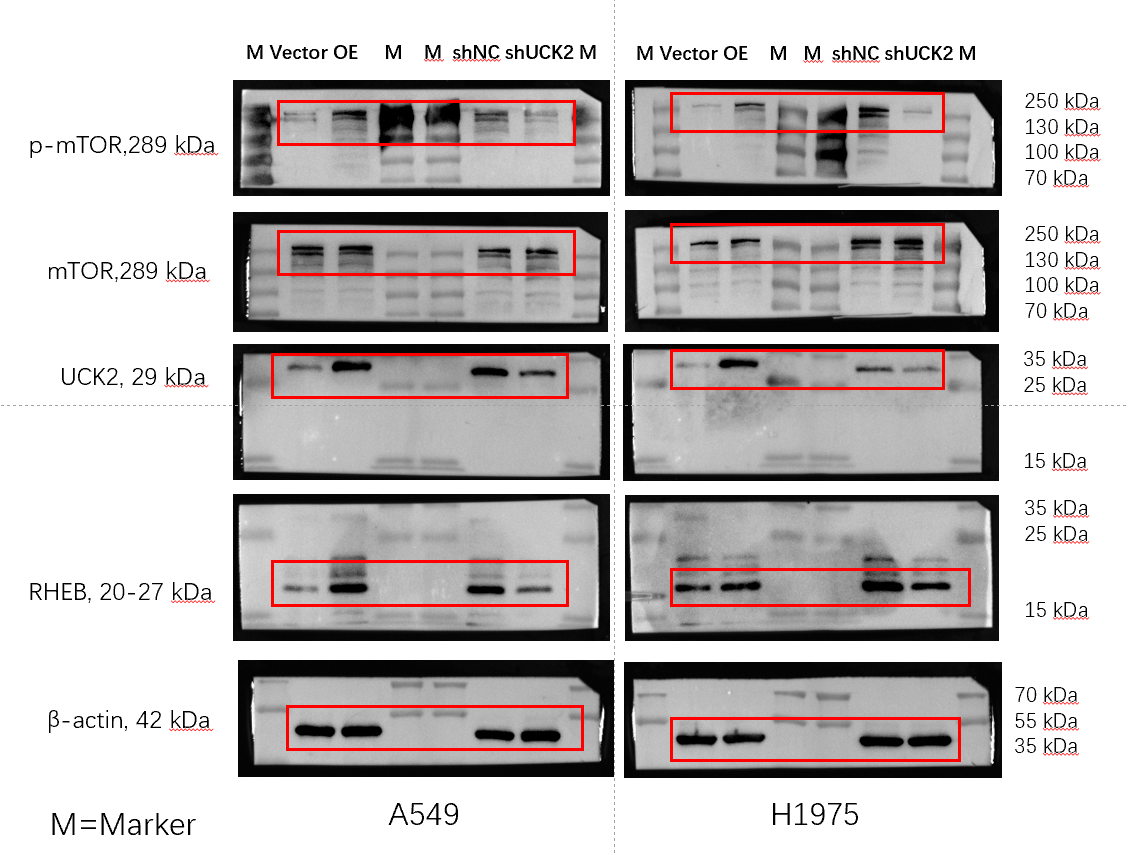


**General Notes**

1. All blots are uncropped, unedited, and retain complete membrane edges + molecular weight markers for traceability.

2. Experimental procedures (e.g., SDS-PAGE, transfer, detection) are detailed in Materials and Methods Section 2.9, 2.11.

3.Antibody Info: See Materials and Methods Section 2.9 & Table A2
